# A Study on the Model of Detecting the Variation of Geomagnetic Intensity Based on an Adapted Motion Strategy

**DOI:** 10.3390/s18010039

**Published:** 2017-12-25

**Authors:** Hong Li, Mingyong Liu, Kun Liu, Feihu Zhang

**Affiliations:** College of Marine Science and Technology, Northwestern Polytechnical University, Xi’an 710072, China; lxglh@mail.nwpu.edu.cn (H.L.); liukunkmz@126.com (K.L.)

**Keywords:** autonomous underwater vehicle, geomagnetic navigation, bio-inspired navigation, gradient descent steering algorithm

## Abstract

By simulating the geomagnetic fields and analyzing the variation of intensities, this paper presents a model for calculating the objective function of an Autonomous Underwater Vehicle (AUV) geomagnetic navigation task. By investigating the biologically inspired strategies, the AUV successfully reaches the destination during geomagnetic navigation without using the priori geomagnetic map. Similar to the pattern of a flatworm, the proposed algorithm relies on a motion pattern to trigger a local searching strategy by detecting the real-time geomagnetic intensity. An adapted strategy is then implemented, which is based on the specific target. The results show the reliability and effectiveness of the proposed algorithm.

## 1. Introduction

Autonomous Underwater Vehicles (AUVs) have been employed in various civil and military fields [1,2], such as ocean data collection, laying pipelines, scouting, and laying mines. However, underwater navigation is still a challenging task for AUVs, in which a trade-off between performance and objective is required [3]. For instance, the Inertial Navigation System (INS) has a horizontal drifting error of less than 2000 m per day, and the cost can reach over one Million CNY [4].

Why do we need geomagnetic navigation when the Global Position System (GPS) is readily available? GPS provides a precise point location, but only measures travel direction when in constant motion. A GPS receiver must collect several sets of latitude and longitude pairs to obtain the direction. In addition, GPS signals may become blocked due to obstructions, adverse terrestrial and space weather, ionospheric conditions, or being underwater or underground. Hence, geomagnetic navigation is an immediate navigational method for air, ground, and water-based systems.

Geomagnetic fields can be treated as a major candidate for providing both the positioning and directional cues [5,6]. Large scale oceanic travelers, such as pelagic birds, are likely to relyon geomagnetic positioning cues during their trips [7]. Aprevious homing experiment showed that the geomagnetic information can help adult green turtles to return to their egg laying sites [8]. Therefore, the geomagnetic fields can be developed asvector fields to provide stable positioning information for an AUV navigation task in this paper.

Geomagnetic navigation technology can provide a reliable navigation reference by measuring geomagnetic fields, with the advantages of concealment, a low cost, and so on [9]. Meanwhile, many geomagnetic sensors have been developed with a much higher sensitivity, such as Mag-03 (0.1 nT) and NS.MS (1 nT). Nowadays, the accuracy of a state-of-the-art magnetometer has reached 0.1 nT [10]. Assisted by the sensors, the geomagnetic navigation technique has also been developed. Most conventional geomagnetic navigation methods for underwater vehicles focus on correlation matching [11,12,13]. However, the performances of the conventional techniques significantly drop when the pre-stored geomagnetic data is missing [14].

To avoid any dependency on a priori geomagnetic data, the bio-inspired geomagnetic navigation method is proposed. The most informative experimental paradigms have verified that animals (like sea turtles and pigeons) can reach their goal locations without using pre-stored geomagnetic data [15,16]. Bionics explains this biological magnetotaxis movement behavior as the response to geomagnetic stimuli. Magnetotaxis is an orientation mechanism which does not determine the gradient direction directly, but which uses the searching strategy to reach the desired target by perceiving the variations in geomagnetic fields potential occurring during the movement. For example, pigeons can always find their home from a distant place where they have never been before [16]. It seems that pigeons just need to perceive the real-time geomagnetic information, whilst being aware of the geomagnetic information of their homes. Therefore, by imitating the animal’s behavior, a new navigation method is proposed by perceiving the variation of the geomagnetic environment. Previous studies have mainly focused on questions about the bio-inspired geomagnetic navigation, and the search algorithm based on the evolutionary strategy was introduced to solve the multi-objective search problem [17,18]. However, the previous algorithm was inefficient and vulnerable to premature convergence under some conditions, and to address this, we will propose an adapted searching algorithm.

This paper presents an Adapted Motion Search Algorithm (AMSA), which utilizes the adaptive local searching mechanism to solve the bio-inspired geomagnetic navigation problem. Here, the pre-stored geomagnetic and geographic information is not required, and the proposed algorithm is based on an adapted motion strategy to search multi-objective geomagnetic components by perceiving the variation of the geomagnetic environment and calculating the values of the objective function at the current position.

The rest of this paper is structured as follows: in Section 2, the biological evidence is given and the magnetotaxis is introduced. In Section 3, the search problem of geomagnetic navigation for an AUV is considered. Next, the adapted algorithm based on the fuzzy logic is explained in Section 4. Then, the simulation setup is introduced in Section 5, followed by an evaluation of the performance. Finally, the conclusion is given in Section 6.

## 2. Biological Evidence

The proposed model is based on the biological evidence, which provides patterns with respect to the navigation ability. Biological studies have demonstrated the existence of magneto-reception in green turtles, homing pigeons, passerine birds, and spiny lobsters [19,20].

**Figure 1:**
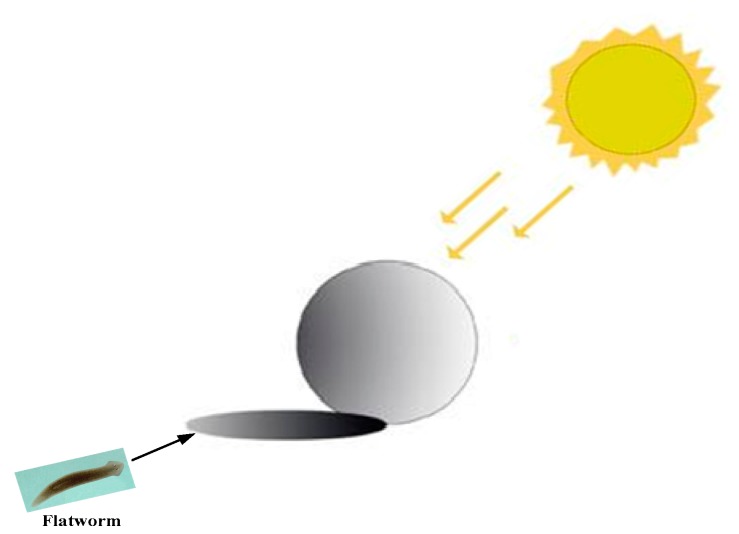
The flatworm moves toward the dark region.

Tropotaxis is a mechanism of the central nervous system of an animal used to guide an estimate for a guidance system, such as Phototaxis, Hygrotaxis, Chemotaxis, and Magnetotaxis, etc. Here, the behavior of the flatworm is an example of phototaxis [21]. It has been shown that the flatworm does not turn directly toward a dark region. Instead, the flatworm increases its turning rate as the light intensity increases [22]. It shows that the flatworm can quickly adjust its direction toward the dark region by perceiving the light intensity of the environment, which is shown in Figure 1.

**Figure 2:**
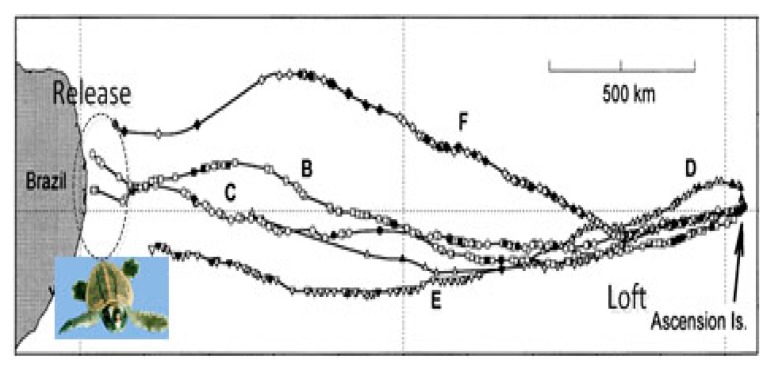
Trajectories of migrating sea turtles.

Magnetotaxis, therefore, is also an orientation mechanism, which is not employed to determine the gradient direction, but to perceive the variations of geomagnetic fields. For example, the recorded paths of the released sea turtles show that the animals can reach their homes without using an a priori geomagnetic map, whilst being aware of the geomagnetic information of their homes, which is shown in Figure 2 [15].

## 3. Problem Formulation

In this paper, the motion of the AUV is considered in the 2D Cartesian coordinate system. This simplification is justified due to the fact that the difference of the geomagnetic in vertical direction is negligible. The kinematic equations of the motion are introduced as follows:

(1) $$\{\begin{array}{c} X_{k}=X_{k-1}+d_{k}\cos(\theta (k-1))\\ Y_{k}=Y_{k-1}+d_{k}\sin(\theta (k-1))\\ \theta (k)=\theta (k-1)+\eta \cdot \Delta \theta (k) \end{array}$$

where k is the time instant (k = 0,1,⋯,N−1), $d_{k}$ is the step size, and θ(k) is the heading of the AUV.

### 3.1. Mathematical Model

**Figure 3:**
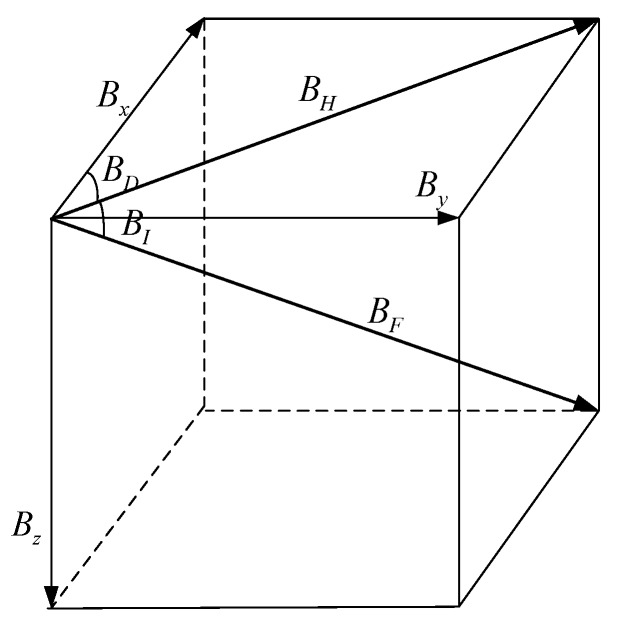
Geomagnetic seven components.

The geomagnetic fields include multiple geomagnetic components [11], which can be described as follows:

(2) $$B=\{B_{1},\cdots ,B_{n}\}$$

where $B_{1},\cdots ,B_{n}$ are defined as the geomagnetic components, such as the north geomagnetic field component $B_{x}$, the east geomagnetic field component $B_{y}$, and the vertical geomagnetic field component $B_{z}$, and the total intensity $B_{F}$, the horizontal magnetic field $B_{H}$, the declination angle $B_{D}$, and the inclination angle $B_{I}$, which are shown in Figure 3.

From the perspective of bionics, biological magnetotaxis behavior is the response to geomagnetic environment stimuli. Its physical significance is that the multiple geomagnetic parameters could determine the geographic locations on the earth [12]. The process of geomagnetic navigation is the convergence process of the geomagnetic components from the current position to the target position. When the geomagnetic components converge to zero, an AUV achieves the navigation task. Therefore, it can be considered as the multi-objective convergence process, as follows:

(3) $$\left\{ \begin{array}{l} \begin{array}{cc} \min & F(\theta (k)) \end{array}\\ \begin{array}{cc} s.t.: & t_{i}=f(B_{i}^{t},B_{i}^{k},\theta (k)) \end{array} \end{array}\right.$$

where $B_{i}^{t}$ represents the geomagnetic components of the target position, $B_{i}^{k}$ represents the geomagnetic components of the current position, and F(·) is the objective function.

### 3.2. General Regularized the Objective Function

The bio-inspired geomagnetic navigation is caused by the magnetotaxis, and the difference error of the geomagnetic components between the current position and the target position is considered as the stimulus. Then, the introduced objective function can describe the difference error of the geomagnetic components, wherein the objective function of the *i*-th geomagnetic component is followed as:

(4) $$f_{i}(\theta (k))={\left(B_{i}^{t}-B_{i}^{k}\right)}^{2},i\in n$$

Considering the different magnitude and unit of the geomagnetic components, the objective function is normalized as follows:

(5) $$F(k)=\frac{1}{N}\sum_{i=1}^{N}\frac{f_{i}(B,k)}{f_{i}(B,0)}=\frac{1}{N}\sum_{i=1}^{N}\frac{{\left(B_{i}^{t}-B_{i}^{k}\right)}^{2}}{{\left(B_{i}^{t}-B_{i}^{o}\right)}^{2}}$$

where $B^{o}=(B_{1}^{o},B_{2}^{o},\cdots ,B_{n}^{o})$ are the geomagnetic components of the starting position.

**Figure 4:**
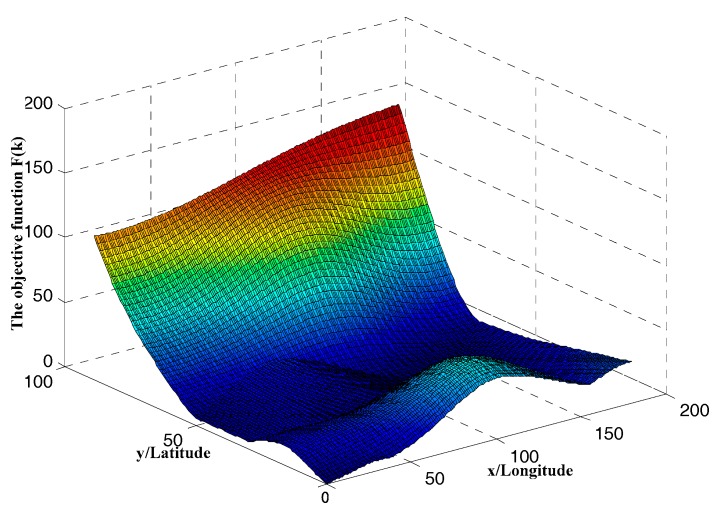
The amplitude characteristic of the objective function.

Figure 4 shows the amplitude characteristic of the objective function F(k). Thais means that the geomagnetic fields area concave and convex non-uniform field.

The purpose is that the objective function converges to the optimal value in the search process, and the vector θ(k) is bound to the optimal vector $\theta^{*}$, which can be expressed as:

(6) $$\underset{k\to \infty }{\lim}F(\theta (k))\to 0$$

Based on the above description, the bio-inspired geomagnetic navigation problem of an AUV could be generalized as the multi-objective searching problem, by using the objective function to explore and exploit the geomagnetic information.

## 4. Method

### 4.1. Search Strategy of AMSA

To balance the exploration and the exploitation, the searching algorithm based on an adapted motion strategy is presented.

Magnetotaxis is an efficient orientation mechanism, which does not determine the gradient direction directly, but perceives the variations in the geomagnetic fields. This is a single-point search method, and each time it generates a new solution from a current solution. The current solution updated with the real-time geomagnetic data is called the iteration solution, and the corresponding search process is called the iteration search. In consideration of the non-uniform geomagnetic fields, the vehicle does not turn directly toward the target region. Instead, the vehicle increases its turning angle when the intensity increases in an effort to arrive at the preferred region.

(1) Heading initialization

By randomly generating the initial heading space *Q*, which is defined as $Q=\{\theta_{j}|j=1,2,\cdots ,n\}$, $\theta_{j}$ can be expressed as:

(7) $$\theta_{j}=\theta_{0}\times j,j\in [1,n]$$

where $\theta_{0}$ is the sampling interval, n is the number of the neighborhood of the current position, and $n=\frac{2\pi }{\theta_{0}}$.

So, when k=1, the initial heading can be selected as follows:

(8) $$\theta (1)=\mathrm{rand}\left\{\theta_{j}\right\}$$

(2) Reactive rules

**Figure 5:**
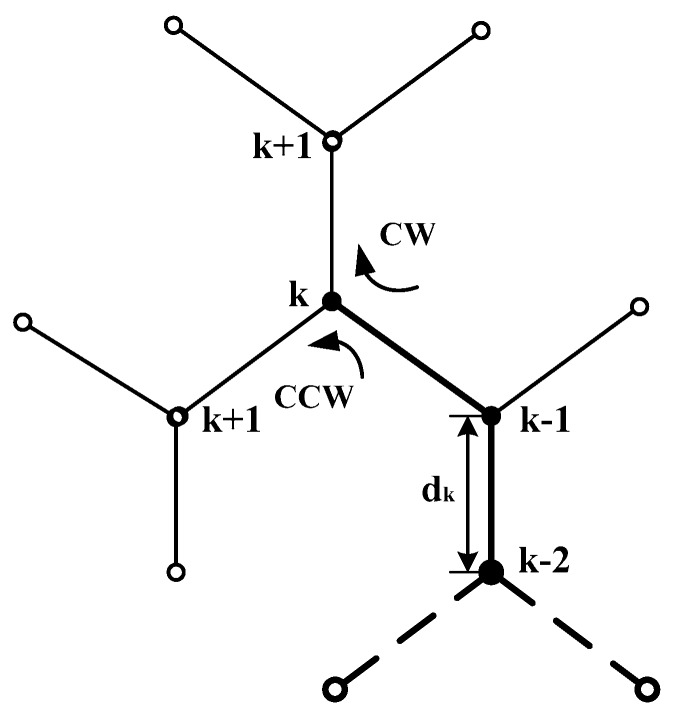
The choice of the turning direction.

In Figure 5, the variation rate of the intensity has been calculated at point (k−2) and (k−1). If the variation at (k−1) has decreased, then the target position close to the right, and the turning of Δθ(k) clockwise (CW) is performed, and η=−1. Otherwise, the turning of Δθ(k) counterclockwise (CCW) is performed, and η=+1.

The choice of the turning direction is explained in the following description of the algorithm:

**Algorithm 1: The choice mechanism of the turning direction**  **repeat** {  **If** (F(k−2)>F(k−1) and the rotation direction is anticlockwise (CCW) at k−1  **or**  F(k−2)≤F(k−1) and the rotation direction is clockwise (CW) at k−1)  **Then** rotate CW Δθ(k) and move forward $d_{k}$  **Else** rotate CCW Δθ(k) and move forward $d_{k}$     }

Here, $d_{k}$ is the step size, where the turning angle is determined by the variation of the intensity. Therefore, Δθ(k) can be expressed as the following:

(9) $$\Delta \theta (k)=\{\begin{array}{cc} 30^{\circ }, & 0<\left|F(k)\right|\leq \gamma_{1}\\ \frac{\left|F(k)\right|-\gamma_{1}}{\gamma_{2}-\left|F(k)\right|}\left(\theta (k-1)-\theta (k-2)\right), & \gamma_{1}<\left|F(k)\right|\leq \gamma_{2}\\ 60^{\circ }, & \gamma_{2}\leq \left|F(k)\right| \end{array}$$

where $\gamma_{1}$ and $\gamma_{2}$ are the experiential thresholds.

(3) Terminate condition

If the searching algorithm meets the termination condition, which can be expressed as:

(10) |F(k)−F(k−1)|<ε

where ε is the small positive constant, then the searching algorithm will terminate; otherwise, go to step (2) above.

**Figure 6:**
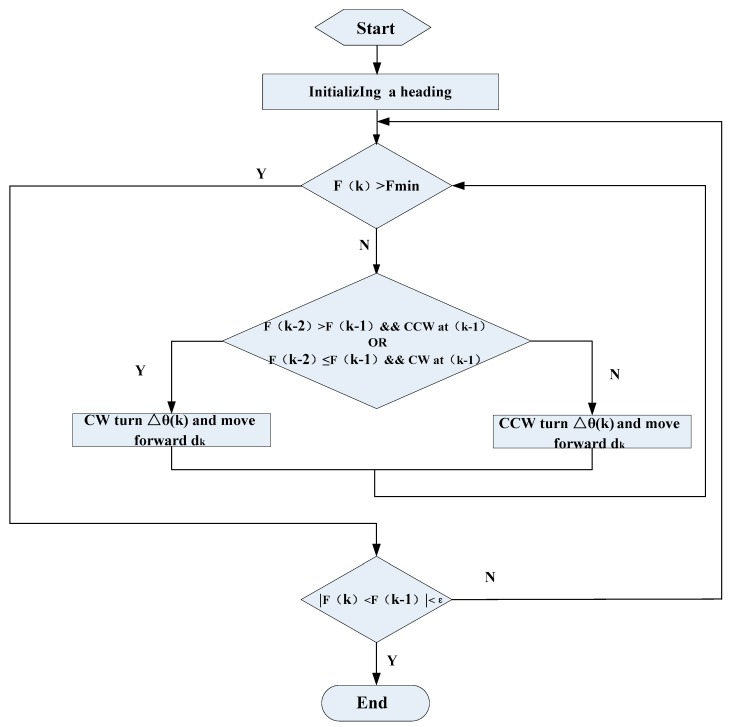
Flow diagram of the searching algorithm.

The proposed search strategy of AMSA is shown in Figure 6.

### 4.2. Online Estimation of Measurement Noise

An online estimation of the measurement noise is performed for the received signal by the geomagnetic sensor. The process is represented as

(11) $$\rho_{i}=\frac{1}{m}\sum_{j=1}^{m}\left|g_{i}(a_{j})-0.5\left(g_{i}(a_{j-1})+g_{i}(a_{j+1})\right)\right|$$

where $\rho_{i}$ is the variable of the measured magnetic data *k*-th, in particular, $\rho_{i}=\left|g_{i}(S(k-1))-g_{i}(S(k))\right|$ when dis(S(k−1),S(k))≤l; $a_{j}$ is the sampling point, $a_{1}=S(k-1)$, and $a_{m}=S(k)$; $g_{i}(\cdot )$ is the measured magnetic data at the sampling point $a_{j}$; $g_{i}(S(k))$ is the measured magnetic variable at S(k); and the actual value of the magnetic variable will be in the interval $\left[g_{i}(S(k))-\rho_{i}(k),g_{i}(S(k))+\rho_{i}(k)\right]$.

Therefore, the estimation of the measurement noise is expressed as

(12) $${\bar{g}}_{i}(S(k))=\left[g_{i}(S(k))-\rho_{i}(k),g_{i}(S(k))+\rho_{i}(k)\right]$$

**Figure 7:**
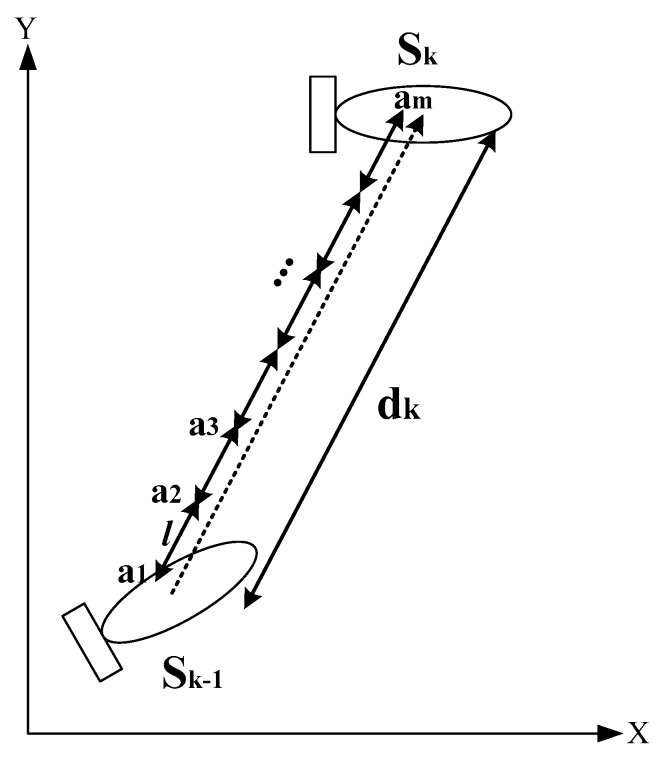
Online estimation of measured noise.

Here, the outlier eliminating is designed to get rid of gross errors when the measured magnetic data goes out of scope. The process is shown in Figure 7.

## 5. Results

To verify the effectiveness of the proposed search algorithm, numerical simulations were implemented.

### 5.1. Simulation Setup

The Word Magnetic Model (WMM2010) is used to provide thereal time geomagnetic data as the geomagnetic sensor [23]. Simulations have been carried out based on the three physical fields: the north magnetic field $B_{x}$, the east magnetic field $B_{y}$, and the total intensity $B_{z}$, which are mutually independent variables and retrieved in real time from the WMM2010.

**Figure 8:**
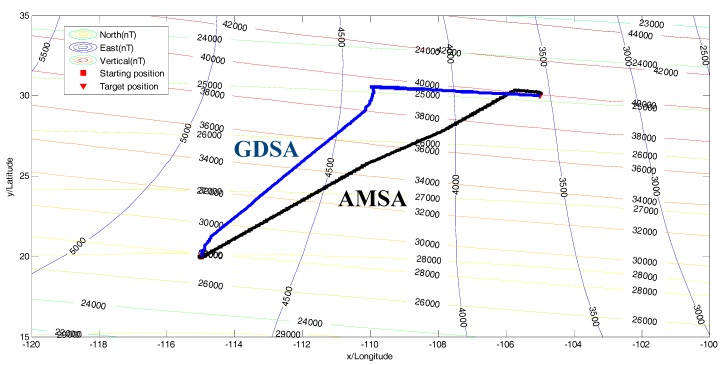
The search paths of two algorithms.

In the simulations, we choose a rectangular area from 15° north latitude and 120° west longitude (15 N, 120 W) to 35° north latitude and 100° west longitude (35 N, 100 W). In this scenario, the starting position is located at (20 N, 115 W), and the goal is located at (30 N, 105 W), where the three geomagnetic components for the starting positions are given as:

$$\left(B_{x}^{k}\;=28034\;\mathrm{nT},\;B_{y}^{k}=474.3\;\mathrm{nT},\;B_{z}^{k}=39764\;\mathrm{nT}\right)$$

the target position is

$$\left(B_{x}^{t}=\;24881\;\mathrm{nT},\;B_{y}^{t}=3557.9\;\mathrm{nT},\;B_{z}^{t}\;=\;47196\;\mathrm{nT}\right)$$

where the red square “□” standsfor the starting position and the red triangle “▽” stands for the target position, which are depicted in Figure 8. Here, the parameter settings are listed in Table 1.

### 5.2. Simulation Results

To demonstrate the effectiveness and efficiency of the proposed algorithm, this article gives a comparison of the different methods.

Comparative studies between the proposed algorithm (AMSA) and the gradient descent steering algorithm (GDSA) were performed to evaluate the effectiveness of the proposed method.

As presented in Figure 8, both strategies can accomplish the navigation task. The input of both the algorithms relied on the environmental stimuli, which was the variation of the objective function. However, the GDSA has the worst performance during the whole space, which is caused by the gradient descent method. But, the AMSA can balance the exploration and the exploitation by perceiving the variations in geomagnetic fields with a better performance.

**Figure 9:**
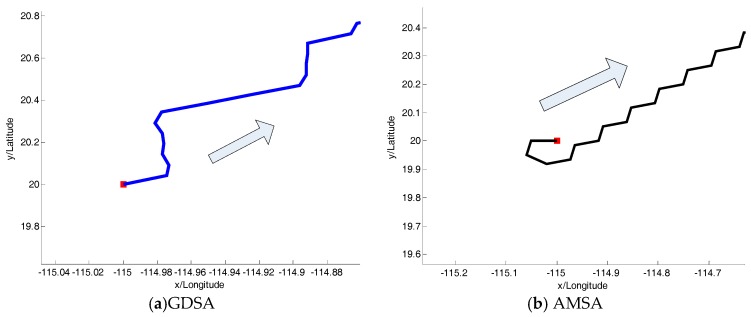
The beginning of two algorithms.

The beginning of two algorithms is shown in Figure 9a,b, respectively. Although the heading in Figure 9b is far away from the target shown above, it does not affect the results of the searching algorithms.

### 5.3. Performance Evaluation of the Algorithms

#### 5.3.1. Convergence Analysis

**Figure 10:**
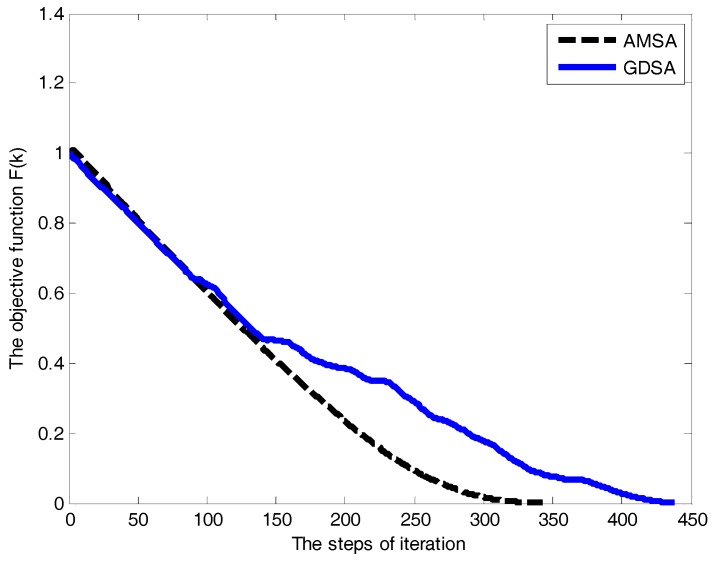
The convergence curves of the objective function.

Figure 10 illustrates the convergence property of the algorithms. The convergence curves of the objective function show a significant difference between the two algorithms. The total numbers of the iteration are 438 and 343, respectively. This shows that the AMSA has a better real-time performance, in contrast to the GDSA algorithm, and can improve the searching efficiency by 22%.

#### 5.3.2. Robustness

**Figure 11:**
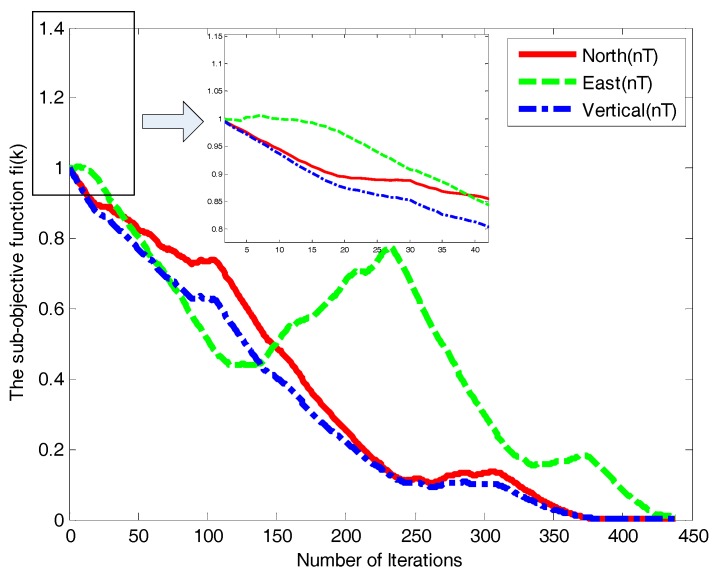
Three components convergence curves of the GDSA.

**Figure 12:**
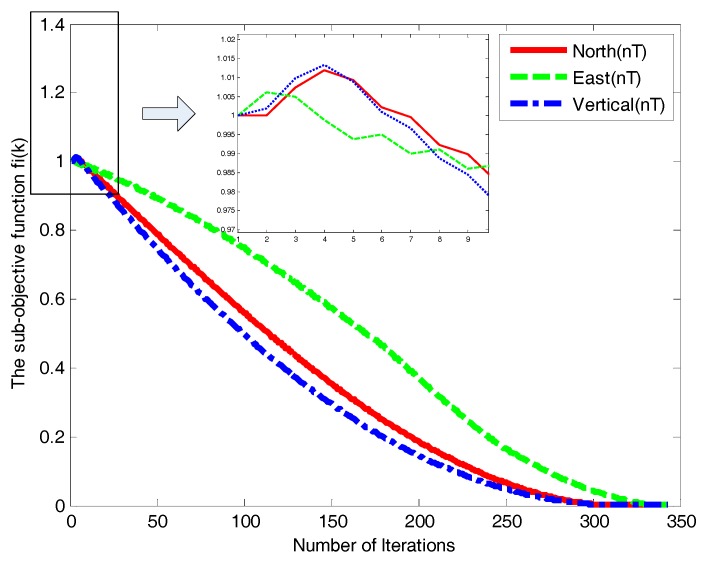
Three components convergence curves of the AMSA.

Figure 11 and Figure 12 illustrate the states of the three geomagnetic components. It can be seen that the convergence of asynchronous consistency occurred in GDSA, whereas AMSA is effective with its rapid convergence and strong robustness. The AMSA is thus more efficient and reliable than GDSA.

#### 5.3.3. Analysis Paths

The straightness of a path describes the amount of turning in a given searching space. It is useful to measure the straightness of the observed paths, in order to evaluate the performance of the algorithm.

The straightness index is a relative measure, which compares the overall displacement G of a path with the total path length T [24,25]. If the path starts at location $\left(x_{0},y_{0}\right)$ after k steps with lengths $d_{k}(k=1,2,\cdots ,K)$, and ends at $\left(x_{t},y_{t}\right)$, then the straightness index τ is given by

(13) $$\tau =\frac{G}{T}=\frac{\left|\left(x_{0},y_{0}),(x_{t},y_{t}\right)\right|}{\sum_{k=1}^{K}d_{k}}$$

This number lies between 0 and 1, where 1 corresponds to the movement in a straight lineand 0 corresponds to a random walk. Therefore, the straightness index is intuitively easy to understand and is also simple to compute. As seen in Table 2, the straightness index of the AMSA is greater than the GDSA, which means that the path for AMSA is smoothing, with respect to a fast convergence speed.

### 5.4. Discussions

The simulations show that the navigation task can be successfully carried out by the magnetotaxis, which involves no determination of the gradient direction, but only the measurement of the variation in the geomagnetic fields. In the simulation, a prior knowledge of geomagnetic information is not required, and the navigation task is solved, only by relying on the three axial magnetic sensor to ascertain the values of geomagnetic components. Therefore, the task of the long-distance navigationcan be practically realized.

Considering that the geomagnetic fields include multiple geomagnetic components and the objective function is a non-uniform field, it is difficult to determine the optimal gradient direction without using an a priori geomagnetic map. In this paper, the gradient descent steering algorithm (GDSA) is used to accomplish the navigation task, by measuring geomagnetic information about the multi-point, which is the neighborhood of the current position, to calculate the objective function of the multi-point and obtainthe optimal gradient direction. In comparison, AMSA is a single-point search strategy without using an a priori geomagnetic map to balance the global exploration and the local exploitation.

The time complexity of the proposed algorithm is O(*k*), but the time complexity of GDSA is O(*k^n^*), where n is the number of the neighborhood of the current position.

In addition, a comparison of time-consuming between the GDSA and the AMSA demonstrates that the presented method can improve the efficiency markedly by 22%.

Therefore, the gradient descent steering algorithm (GDSA) is not suitable to be used in the realistic navigation application.

## 6. Conclusions

The paper presents an active searching strategy based on the magnetotaxis, which is derived from a biological-inspired tropotaxis behavior, for long-range navigation of an Autonomous Underwater Vehicle (AUV). It is a novel method without using the priori geomagnetic information. Firstly, the geomagnetic navigation problem can be attributed to a multi-objective searching problem. Then, the geomagnetic navigation modelis established by constructing an objective function. Meanwhile, two algorithms are presented and compared to test the effectiveness of the proposed method. Finally, the results indicate that the AMSA is more efficient than the GDSA.

Furthermore, by taking the complex and unknown environments for an AUV into consideration, we will investigate the spatial discrimination of the searching algorithm in the presence of the geomagnetic anomaly environments.

## Figures and Tables

**Table 1:** Setting navigation parameters.

No	Parameters	Size
1	$\theta_{0}$	30°
2	$\gamma_{1}$	0.1
3	$\gamma_{2}$	0.3
4	ε	0.0001
5	$F_{\min}$	0.05
6	$d_{k}$	500 m
7	σ	10

**Table 2:** A comparison between two algorithms.

Algorithm	The Steps of Iteration	The Straightness Index
GDSA	438	0.6217
AMSA	343	0.7514
